# Disentangling the innate immune responses of intestinal epithelial cells and lamina propria cells to *Salmonella* Typhimurium infection in chickens

**DOI:** 10.3389/fmicb.2023.1258796

**Published:** 2023-10-03

**Authors:** Kate Sutton, Tessa Nash, Samantha Sives, Dominika Borowska, Jordan Mitchell, Prerna Vohra, Mark P. Stevens, Lonneke Vervelde

**Affiliations:** ^1^Division of Immunology, The Roslin Institute, Royal (Dick) School of Veterinary Studies, University of Edinburgh, Edinburgh, United Kingdom; ^2^Institute for Immunology and Infection Research, School of Biological Sciences, University of Edinburgh, Edinburgh, United Kingdom; ^3^Division of Bacteriology, The Roslin Institute, Royal (Dick) School of Veterinary Studies, University of Edinburgh, Edinburgh, United Kingdom

**Keywords:** intestine, enteroid, innate immunity, *Salmonella*, Typhimurium

## Abstract

*Salmonella enterica* serovar Typhimurium (STm) is a major foodborne pathogen and poultry are a key reservoir of human infections. To understand the host responses to early stages of *Salmonella* infection in poultry, we infected 2D and 3D enteroids, the latter of which contains leukocytes, neurons, and mesenchymal cells that are characteristic of the lamina propria. We infected these enteroids with wild-type (WT STm), a non-invasive mutant lacking the *prgH* gene (Δ*prgH* STm), or treated them with STm lipopolysaccharide (LPS) and analyzed the expression of innate immune related genes by qPCR at 4 and 8 h. The localization of the tight junction protein, ZO-1, expression was disrupted in WT STm infected enteroids but not Δ*prgH* STm or LPS treated enteroids, suggesting a loss of epithelial barrier integrity. The innate immune response to LPS was more pronounced in 2D enteroids compared to 3D enteroids and by 8 hpi, the response in 3D enteroids was almost negligible. However, when STm adhered to or invaded the enteroids, both 2D and 3D enteroids exhibited an upregulation of inflammatory responses. The presence of lamina propria cells in 3D enteroids resulted in the unique expression of genes associated with immune functions involved in regulating inflammation. Moreover, 2D and 3D enteroids showed temporal differences in response to bacterial invasion or adherence. At 8 hpi, innate responses in 3D but not 2D enteroids continued to increase after infection with WT STm, whereas the responses to the non-invasive strain decreased at 8 hpi in both 2D and 3D enteroids. In conclusion, STm infection of chicken enteroids recapitulated several observations from *in vivo* studies of *Salmonella*-infected chickens, including altered epithelial barrier integrity based on ZO-1 expression and inflammatory responses. Our findings provide evidence that *Salmonella*-infected enteroids serve as effective models for investigating host-pathogen interactions and exploring the molecular mechanisms of microbial virulence although the 3D model mimics the host more accurately due to the presence of a lamina propria.

## 1. Introduction

*Salmonella enterica* are Gram-negative rod-shaped facultative anaerobic bacteria that are comprised of over 2,600 antigenically distinct serovars. *Salmonella enterica* serovar Typhimurium (STm), typically has a broad host range and transmits via contaminated food or water, causing severe gastroenteritis. The consumption of poultry meat and eggs contaminated with STm is a significant contributor to human infections. Intestinal inflammation that characterizes *Salmonella* gastrointestinal infection is caused by the infection of effector proteins into host cells by a Type 3 secretion system (T3SS-1) encoded by *Salmonella* pathogenicity island 1 (SPI-1) (Mills et al., 1995). Effector proteins delivered by T3SS-1 promote bacterial invasion by orchestrating rearrangements in the subcortical actin cytoskeleton and activate inflammatory responses (Raffatellu et al., 2005; Boyle et al., 2006). In mammals, mutations in T3SS-1 genes, such as *prgH*, reduce the ability of STm to colonize the intestine and induce inflammatory and secretory responses (Klein et al., 2000). T3SS-1 contributes to colonization of the avian intestine by STm (Chaudhuri et al., 2013). However, inflammation is less pronounced than in mammals, with STm typically being carried asymptomatically in chickens over 1-week-old and shed persistently in the faeces (Raffatellu et al., 2005). Neonatal chicks are highly susceptible to STm infection, which causes systemic infection and death (Barrow et al., 1987; Withanage et al., 2005). Although adults are less susceptible, STm can colonise the gastrointestinal tract without an associated clinical disease.

*In vivo* studies have provided considerable knowledge about the nature and consequences of mucosal immune responses to STm in the chicken intestine (Withanage et al., 2004, 2005; Iqbal et al., 2005; Fasina et al., 2008; Bescucci et al., 2022). An *in vitro* analysis of Salmonella - host interactions in a system containing all cells, such as epithelial cells and lamina propria cells including leukocytes, glial and mesenchymal cells are lacking. In mammals the interplay between cells relies on co-culture systems with either monocyte or bone marrow-derived mononuclear phagocytes which do not fully encompass the heterogeneity of the intestinal tissue (Noel et al., 2017; Staab et al., 2020). Three-dimensional (3D) intestinal organoids, when derived from primary tissue are known as enteroids, closely mimic the morphology and physiology of the intestine, and are emerging as *in vitro* models to study host-pathogen interactions. Intestinal enteroids grown in an extracellular matrix consist of a central lumen lined by a single layer of polarized epithelial cells with their basolateral surface in contact with the extracellular matrix scaffold (Sato et al., 2009). In contrast to cell lines, enteroids recapitulate all major differentiated epithelial cell lineages, including enterocytes, goblet cells, enteroendocrine cells, Paneth cells, and tuft cells. Zhang et al. (2014) were the first to analyze STm infection in murine enteroids demonstrating epithelial cell invasion, disruption of tight junctions and NFκB related pro-inflammatory responses. Human, bovine and porcine enteroids have since been reported to be susceptible to STm (Zhang et al., 2014; Forbester et al., 2015; Derricott et al., 2019). However, the fully enclosed lumen of mammalian enteroids poses a challenge to deliver the pathogens to the epithelial surface. Recently, apical-out enteroids derived from basal-out human, porcine, bovine and ovine enteroids have been developed (Co et al., 2021; Smith et al., 2021; Blake et al., 2022; Joo et al., 2022). A study has shown that human apical-out enteroids recapitulate specific morphological hallmarks of STm infection in humans including epithelial barrier disruption and cytoskeletal reorganization (Co et al., 2021).

Avian floating 3D enteroids naturally develop in an advantageous apical-out conformation with apical microvilli facing the media and an inner core resembling the lamina propria, containing leukocytes, and mesenchymal and neuronal cells (Nash et al., 2021, 2023). In addition, a chicken 2D enteroid model that self-organizes into an epithelial and mesenchymal sub-layer but lacks the underlying lamina propria cells has been developed (Orr et al., 2021). The aim of this study was to disentangle the innate immune response between a system with (3D) and without (2D) lamina propria cells to STm infection by comparing the gene expression profiles between uninfected and infected enteroids. In addition, we analyzed the effects of an invasion deficient strain, a Δ*prgH* mutant of STm, on the innate immune response in each enteroid model. Our study reveals marked differences in the response to STm infection in both models. Therefore, these models provide valuable insights into deciphering the distinct responses in systems where lamina propria cells are present or absent, such that findings with simpler cell-based models should be interpreted with caution.

## 2. Materials and methods

### 2.1. Animals

Experiments were performed using embryonic day 18 (ED18) Hy-Line Brown fertile embryos (*Gallus gallus*) obtained from the National Avian Research Facility, University of Edinburgh, UK. Embryos were humanely culled under the authority of a UK Home Office Project License (PE263A4FA) in accordance with the guidelines and regulations of the Animals (Scientific Procedures) Act 1986.

### 2.2. Generation of chicken 2D and 3D enteroids

Tissue from duodenum, jejunum and ileum of ED18 chickens were retrieved and placed in phosphate buffered saline (PBS, Mg^2+^ and Ca^2+^ free) until use. For each independent culture, the intestines from five embryos were pooled. For the generation of 3D enteroids, the villi were released from the tissue as previously described (Nash et al., 2021). In brief, intestinal tissue was cut open longitudinally and cut into 3 mm pieces. The tissues were digested with *Clostridium histolyticum* type IA collagenase (0.2 mg/mL, Merck, Gillingham, UK) at 37°C for 50 min with shaking at 200 rpm. Single cells were removed by filtering the digestion solution through a 70 μM cell strainer (Corning, Loughborough, UK). The villi were obtained by rinsing the inverted strainer. The collected villi were centrifuged at 100 *g* for 4 min. The 3D enteroids were seeded at 200 villi per well in 24 well plates with 400 μl of Floating Organoid Media (FOM media); Advanced DMEM/F12 supplemented with 1X B27 Plus, 10 mM HEPES, 2 mM L-Glutamine and 50 U/mL Penicillin/Streptomycin [Thermo Fisher Scientific (TFS), Paisley, UK]. For 2D enteroid generation, freshly isolated intestinal villi were enzymatically digested with Accutase (TFS) as previously described (Orr et al., 2021). To remove the majority of the fibroblasts, cells were resuspended in FOM media supplemented with 1X N2 supplement (TFS), 100 ng/mL human (hu) epidermal growth factor (huEGF, TFS), 10 μM CHIR 99021 (Stratech Scientific), 10 μM Y27632 (Stem Cell Technologies) and 100 nM LDN193189 (Cambridge Bioscience). Cells were incubated for 3 h at 37°C, 5% CO_2_ in 6 well plates. Non-adherent cells were removed, counted and seeded at 2 × 10^5^ cells in uncoated 24 well plates with 350 μl of FOM media supplemented with 1X N2 supplement, 100 ng/mL huEGF, 100 ng/mL huR-spondin, 50 ng/mL huNoggin (R&D Systems), 10 μM CHIR 99021 and incubated at 37°C, 5% CO_2_.

### 2.3. Preparation of *Salmonella*

*Salmonella* Typhimurium strain ST4/74 nal*^R^* (WT) is known to colonize the chicken intestine proficiently (Chaudhuri et al., 2013) and was routinely cultured in Luria-Bertani broth containing 20 μg/mL of naladixic acid (TFS). An isogenic ST4/74 nal*^R^* Δ*prgH:kan* mutant, deficient in bacterial invasion, was additionally cultured in the presence of 20 μg/mL of kanamycin (Merck) and has been described previously (Balic et al., 2019). Both strains were transformed with a plasmid that constitutively expresses green fluorescent protein (GFP), pFVP25.1 (Valdivia and Falkow, 1996; Vohra et al., 2019), which was maintained in the presence of 50 μg/mL of ampicillin (Merck). Bacteria were incubated for 18 h at 37°C with shaking at 180 rpm to an optical density of 1 at 600 nm and pelleted at 2,000 *g* for 10 min. Bacteria were washed twice with PBS and resuspended in 10 mL of PBS. Ten-fold serial dilutions were plated in duplicate on LB agar containing 20 μg/mL of naladixic acid incubated at 37°C overnight to determine viable counts retrospectively.

### 2.4. Bacterial infection and LPS treatment of 2D and 3D enteroids

On day 2 of culture, 3D enteroids were pelleted at 100 *g* for 4 min and reseeded at 200 enteroids per well on 24 well plates (Corning) in 400 μL of FOM media without antibiotics. Similarly, on day 2 of culture, 2D enteroids were washed twice with PBS and cultured for a further 24 h in FOM without antibiotics, CHIR and Y27632. After 24 h, on day 3 of culture, the 3D and 2D enteroids were treated with WT or Δ*prgH* STm strains (2 × 10^5^), LPS (1 ug/mL) derived from STm (product code L6143, Merck) or media only. At 4 and 8 h post-infection (hpi) the supernatant was removed and cells washed with PBS and lysed in RLT Plus buffer (Qiagen) containing 10 μg/mL 2-mercaptoethanol (Merck). For increasing bacterial dose analysis, 3D enteroids were infected with 1 × 10^3^, 500 or 250 CFU of WT STm for 3 h. The 3D enteroids were further homogenized using QIAshredder columns (Qiagen). Samples were stored at −20°C until use.

### 2.5. Immuno-fluorescent staining and microscopy

For immuno-fluorescent staining, chicken 2D enteroids were grown on 2% Matrigel (Corning) coated transwell inserts (VWR, 0.33 cm^2^) in 24 well plates (Orr et al., 2021) while 3D enteroids were grown as outlined above. Chicken 2D and 3D enteroids were treated with WT or Δ*prgH* STm, LPS or media alone as outlined above. At 4 and 8 h post-treatment, cells were gently washed with PBS and fixed with 4% w/v paraformaldehyde (TFS) for 15 min at room temperature and blocked with 5% v/v goat serum in permeabilization buffer (0.5% w/v bovine serum albumin and 0.1% w/v Saponin in PBS; Sigma-Aldrich). Permeabilization buffer was used to dilute all antibodies. Cells were stained with mouse anti-human ZO-1 (Abcam, IgG1, clone A12) overnight at 4°C followed by the secondary antibody, goat anti-mouse IgG1 Alexa Fluor^®^594 (TFS) for 2 h on ice. Cells were counterstained with Hoechst 33,258 and Phalloidin Alexa Fluor^®^647 (TFS) to stain for nuclei and F-actin, respectively. Slides were mounted using ProLong™ Diamond Antifade medium (TFS). Controls comprising of secondary antibody alone were prepared for each sample. Images and Z-stacks were captured using an inverted LSM880 (Zeiss) with 40X and 63X oil lenses using ZEN 2012 (Black Edition) software and were analyzed using ZEN 2012 (Blue Edition). Z-stack modeling was performed using IMARIS software (V9).

### 2.6. Isolation of RNA and reverse transcription

Total RNA from the enteroids was extracted using an RNeasy Plus Mini Kit (Qiagen) consisting of a genomic DNA column eliminator according to manufacturer’s instructions and quantified spectrophotometrically. Five independent 3D enteroids samples and three independent 2D enteroids samples that were of high quality were used for qPCR analysis (RNA concentration of > 100 ng and a 260/230 ratio of 2). Reverse transcription was performed using the High Capacity Reverse Transcription Kit (Applied Biosystems) according to manufacturer’s instructions with random hexamers and oligo (dT)18, containing 100 ng of total RNA. The cDNA samples were stored in −20°C until use.

### 2.7. Pre-amplification and quantitative PCR using 96.96 Integrated Fluid Circuits dynamic array

Pre-amplification of cDNA was performed as previously described (Borowska et al., 2019; Bryson et al., 2023). In brief, 2.5 μl of a 200 nM stock pool of each primer pair (Supplementary Material 1) was added to 5 μl of TaqMan PreAmp Master Mix (Applied Biosystems) and 2.5 μl of 1:5 dilution of cDNA. Due to its high level of expression, the ribosomal 28S (r28S) primer pair were excluded from the stock primer mix. Samples were incubated at 95°C for 10 min followed by 14 cycles of 95°C for 15 s and 60°C for 4 min. Unincorporated primers were digested from the pre-amplified samples using 16 U/μl Exonuclease I (*E. coli*, New England Biolabs) at 37°C for 30 min. High-throughput qPCR was performed with the microfluidic 96.96 Dynamic array (Standard BioTools UK) as previously described (Borowska et al., 2019; Bryson et al., 2023). Each sample was run in duplicate with 89 target genes and 5 reference gene primers. In order to reduce inter-plate variation, an inter-plate calibrator (IPC) sample was employed on each array. The IPC sample comprised of pre-amplified cDNA derived from splenocytes stimulated with Concanavalin A (10 μg/mL, Sigma-Aldrich) for 4 h. Quantitative PCR was performed on the BioMark HD instrument (Fluidigm) using the thermal cycling conditions as previously reported (Borowska et al., 2019). The fluorescence emission was recorded after each cycling step. Raw qPCR data quality threshold was set to 0.65-baseline correction to linear (derivative) and quantitation cycle (Cq) threshold method to auto (global) using the Real-Time PCR Analysis software 3.1.3 (Fluidigm).

### 2.8. RT-qPCR data analysis

Data pre-processing, normalization, relative quantification and statistics were performed using GenEx6 and GenEx Enterprise (MultiD Analyses AB). The qPCR performance base-line correction and set threshold of the instrument was compensated across the two array runs using the IPC samples. Delta Ct values were obtained by normalizing the Ct values of the target genes with the geometric mean of three reference genes, GAPDH, TBP and r28S, identified from a panel of five references using NormFinder. The technical repeats were averaged and relative quantities were set to the maximum Cq value for a given gene. Relative quantities were log transformed (log2) and differentially expressed genes (DEG) between uninfected and infected enteroids was calculated by the −2^–ΔΔCT^ method (Livak and Schmittgen, 2001). Principal component analysis was performed using *prcomp* function and plots were generated using *ggplot* in RStudio (V1.1.442).

### 2.9. Statistical analysis

Statistical analysis of the differentially expressed genes (DEGs) between control (untreated) and LPS treated or between control and STm infected 2D or 3D enteroids was performed using GENEx5 and GenEx Enterprise (MultiD Analyses AB). Correction for multiple testing was performed with the estimation of false discovery rate using Dunn-Bonferroni correction threshold of 0.00054. After correction, DEG with a significant difference (*P* < 0.05) and a fold change of ≥ 1.5 were identified. Finally, for each comparison, the fold change of untreated and treated samples at their respective timepoint was calculated. Graphs were prepared using GraphPad Prism 9.

## 3. Results

### 3.1. *Salmonella* Typhimurium alters tight junctions in chicken enteroids

**FIGURE 1:**
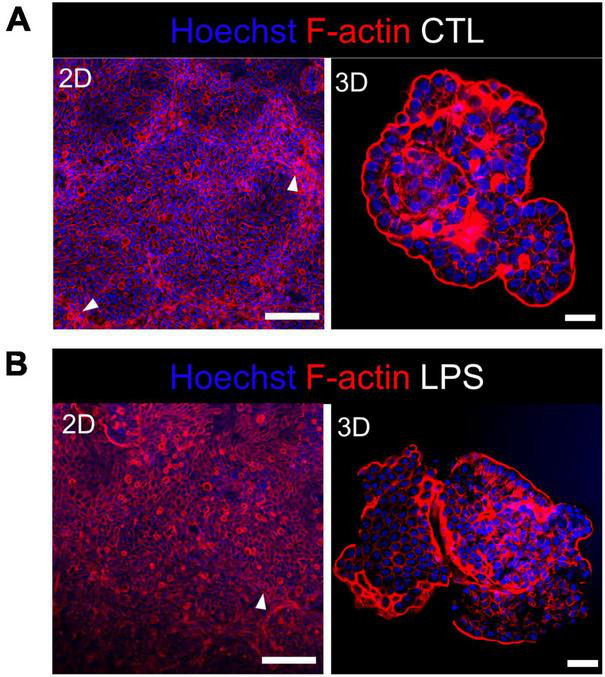
Untreated and LPS-treated 2D and 3D enteroids exhibit unaltered organization of F-actin. Confocal micrographs of F-actin organization in **(A)** untreated or **(B)** LPS-treated 2D and 3D enteroids. Chicken 2D enteroids were cultured on Matrigel coated transwell inserts and images were retrieved using 20X objective. In one planar view, both epithelial cells and the underlying mesenchymal cells with long F-actin filaments can be observed (white arrow). Images are representative of 3 independent experiments. Scale bars = 200 μm (2D) and 100 μm (3D).

**FIGURE 2:**
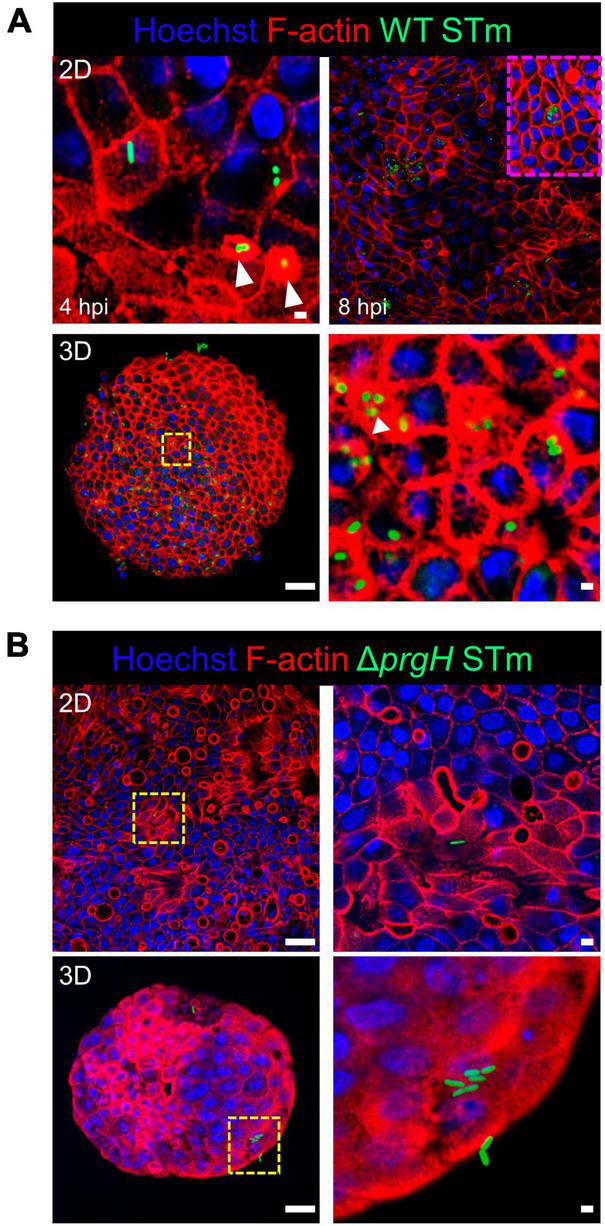
WT STm infection remodels F-actin in chicken enteroids. Confocal micrographs showing F-actin remodeling in **(A)** WT STm infected 2D and 3D enteroids. At 4 hpi, dense F-actin staining can be observed surrounding the invading bacteria consistent with reorganization of subcortical actin stimulating membrane ruffling (white arrows). By 8 hpi, numerous bacteria can be observed within epithelial cells. At 8 hpi the number of internalized bacteria was markedly higher in 3D enteroids inoculated with the same bacterial dose. **(B)** No F-actin remodeling was observed in Δ*prgH* STm infected 2D and 3D enteroids at 8 hpi, although bacteria were observed in close association with the apical surface of epithelial cells. Images are representative of 3 independent experiments. Scale bars = 100 μm and 50 μm.

We studied the early response of chicken 2D and 3D enteroid cultures to infection with STm in order to understand the relative contribution of epithelial and lamina propria cells to the response. In untreated and LPS-treated 2D and 3D enteroids actin expression was evenly distributed between cells and at the apical surface (Figures 1A, B). Images taken using a low objective, show the non-planar surface of 2D enteroids and demonstrates that in a certain planar view, both the epithelial and the underlying mesenchymal cells, with long F-actin filaments, can be observed (Figures 1A, B). At 4 hpi with WT STm, raised F-actin structures were observed around the bacteria at the apical surface (Figure 2A). Z-stack modeling of 2D enteroids demonstrates punctuated F-actin expression on the apical surface of a WT STm infected cell (Supplementary Video 1). At 8 hpi, bacteria could be detected within individual cells in 2D enteroids (Figure 2A). Despite the same inoculum used in WT STm infected 3D enteroids, we observed more remodeled F-actin around invading bacteria (Figure 2A). In contrast, no actin remodeling was observed in Δ*prgH* STm infected 2D and 3D enteroids although on occasion, bacteria were observed in close proximity to the apical surface of epithelial cells (Figure 2B) or found trapped in the folds of the epithelial buds (Supplementary Figure 1). This is consistent with the known role of T3SS-1 in promoting membrane ruffling and invasion.

**FIGURE 3:**
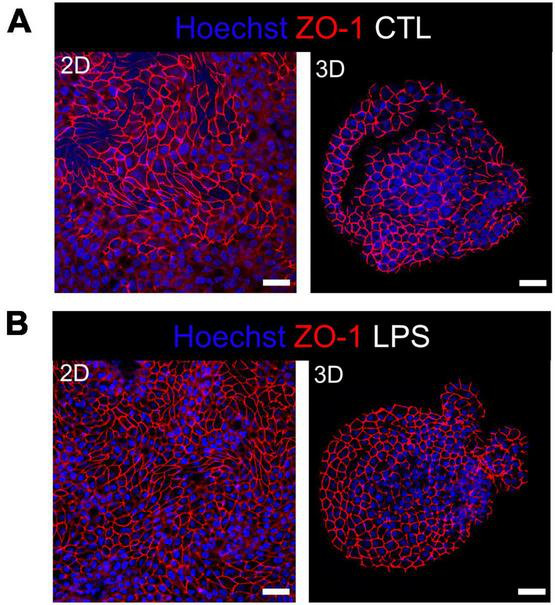
Distribution of the tight junction protein ZO-1 at cell-cell junctions in untreated and LPS treated enteroids. Confocal micrographs of ZO-1 distribution in **(A)** untreated or **(B)** LPS treated 2D and 3D enteroids show typical ZO-1 distribution at 8 h. Images are representative of 3 independent experiments. Scale bars = 100 μm.

**FIGURE 4:**
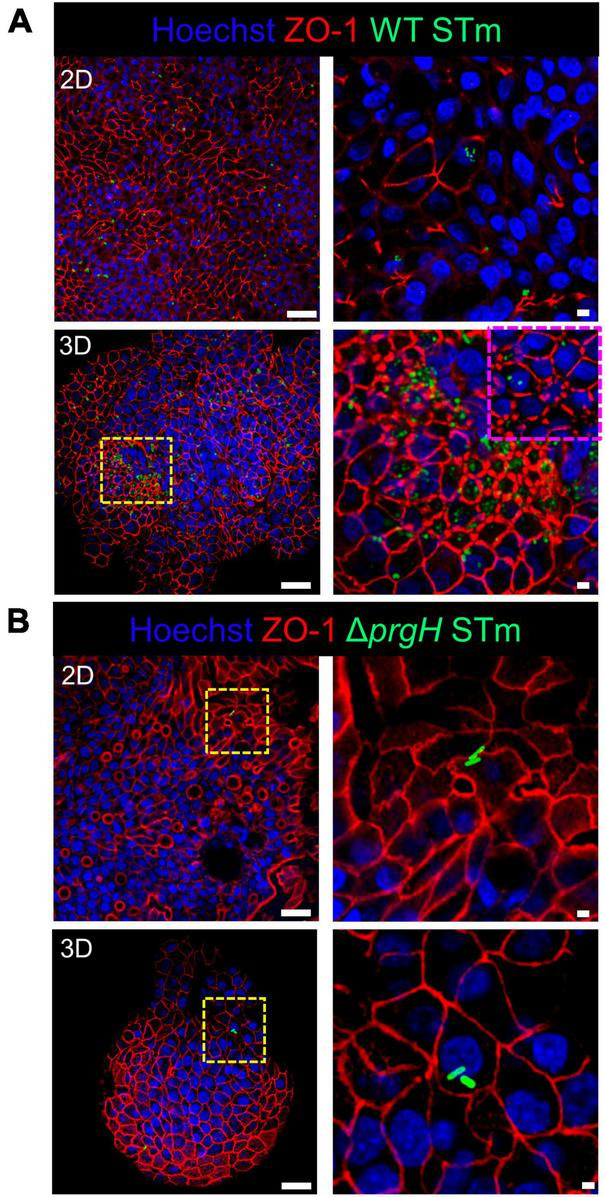
WT STm but not Δ*prgH* STm infection alters the distribution of the tight junction protein ZO-1 in chicken enteroids. Confocal micrographs showing the distribution of ZO-1 in **(A)** WT STm infected 2D and 3D enteroids, which leads to reduced ZO-1 expression in 2D enteroids and altered ZO-1 distribution in 3D enteroids at 8 hpi (yellow/magenta dash insert images). **(B)** ZO-1 distribution were unaltered in Δ*prgH* STm infected 2D and 3D enteroids at 8 hpi. Images are representative of 3 independent experiments. Scale bars = 100 μm and 50 μm.

Integrity of the epithelial barrier was analyzed by immuno-fluorescent staining of the tight junction protein ZO-1. In untreated and LPS treated 2D and 3D enteroids, ZO-1 expression was localized to the lateral membrane, showing a typical polygonal shape of enterocytes and demonstrates that LPS had no effect on ZO-1 localization (Figures 3A, B). No difference in the localization of ZO-1 was observed at 4 hpi across each model after WT or Δ*prgH* STm infection (Supplementary Figure 2). At 8 hpi, ZO-1 localization became discontinuous in WT STm infected 2D enteroids (Supplementary Video 2). In addition to being discontinuous, ZO-1 in 3D enteroids formed dense strands, which was not observed in 2D enteroids following infection. In addition to being discontinuous, ZO-1 in 3D enteroids formed dense strands, which was not observed in 2D enteroids following infection (Figure 4A). The Δ*prgH* STm strain did not elicit changes in ZO-1 distribution, which resembled that observed in the untreated 2D and 3D enteroids (Figure 4B). Occasionally, the Δ*prgH* STm strain was observed in close proximity to the apical surface of epithelial cells, but it did not cause any disruption to ZO-1 localization (Supplementary Video 3).

### 3.2. Global transcriptional profiles cluster by treatment and culture model

**FIGURE 5:**
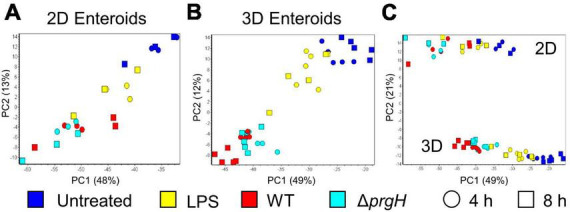
Principal component analysis (PCA) of gene expression profiles. PCA analysis of untreated, LPS treated, and WT and Δ*prgH* STm infected **(A)** 2D enteroids (*n* = 3) and **(B)** 3D enteroids (*n* = 5) at 4 hpi and 8 hpi alone and **(C)** with both datasets.

To disentangle the innate immune responses of 2D and 3D chicken enteroids to *Salmonella* or its LPS, the mRNA expression levels of 89 innate-immune related genes were analyzed using Fluidigm Biomark high-throughput qPCR at 4 and 8 h post-treatment. To assess the degree of heterogeneity between replicates and treatments, the global transcriptional profiles were compared using Principal Component Analysis (PCA). This analysis demonstrated that sample clustering was primarily by treatment as the uninfected and infected enteroids segregated from each other along the first principal component (PC1, Figures 5A, B). This difference accounted for 48–49% of the total variance in 2D and 3D enteroids, respectively, and suggested that treatment is a greater determinant of transcriptional variance rather than time post-infection (PC2). PCA of the total dataset shows sample clustering based on the culture system, 2D versus 3D (Figure 5C, PC2), corresponding to the presence of different cell types such as immune cell in 3D enteroids while the effect of treatment (PC1) was preserved.

### 3.3. Chicken 2D enteroids exhibit a more pronounced innate immune response to *Salmonella* LPS than 3D enteroids

**FIGURE 6:**
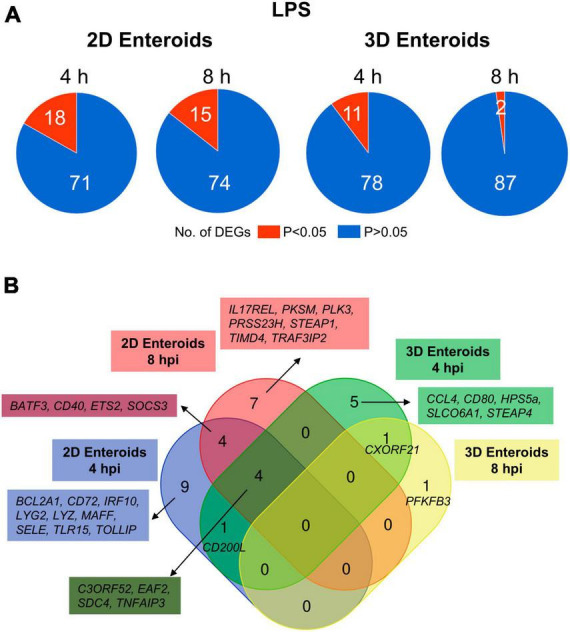
Chicken 2D enteroids exhibit more pronounced responses to LPS treatment. **(A)** Pie charts indicating the number of statistically significant (*P* < 0.05) and non-significant (*P* > 0.05) DEGs in LPS treated 2D enteroids (*n* = 3) and 3D enteroids (*n* = 5) at 4 h and 8 h compared to time-matched, respective controls. DEGs with a significant difference (*P* < 0.05) and a fold change of ≥ 1.5 were identified. **(B)** Venn diagram showing common and unique DEGs across each enteroid model and timepoint.

Chicken 2D and 3D enteroids were treated with STm-derived LPS for 4 and 8 h. Firstly, the number of statistically differentially expressed genes (DEGs) with a fold change ≥ 1.5 at *P* < 0.05 compared to their respective time-matched controls was analyzed (Figure 6A). The number of DEGs was higher at 4 and 8 h in the 2D compared to 3D enteroids and the number of DEG substantially decreased with time in the 3D enteroids compared to the 2D enteroids. Next, the commonality and difference in the genes regulated in 2D and 3D enteroids were compared (Figure 6B). There was no core set of common DEGs between 2D and 3D enteroids at 4 and 8 h post-LPS treatment. Of the 11 DEGs regulated by 3D enteroids at 4 h post-LPS treatment, four were common to 2D enteroids, *C3ORF52*, *TNFAIP3*, *EAF2*, and *SDC4*. DEGs specifically regulated in 2D enteroids were related to TLR signaling (*TOLLIP, TRAF3IP2, TLR15*), effector protein functions (*LYZ*, *LYG2*) and regulation of immune responses (*BATF3, CD200L, IRF9, IL17REL, SOCS3*).

The mRNA expression levels of a majority of the DEGs increased by 2-7-fold at *P-*values of less than 0.05 in LPS treated enteroids compared to untreated enteroids except for *SELE* and *TIMD4*, which decreased in expression in 2D enteroids (Supplementary Material 2). In conclusion, *Salmonella*-derived LPS treatment of chicken 2D and 3D enteroids resulted in differential innate immune responses, with 2D enteroids having a more pronounced response compared to 3D enteroids.

### 3.4. 2D and 3D enteroids have differing temporally responses to *Salmonella* infection

**FIGURE 7:**
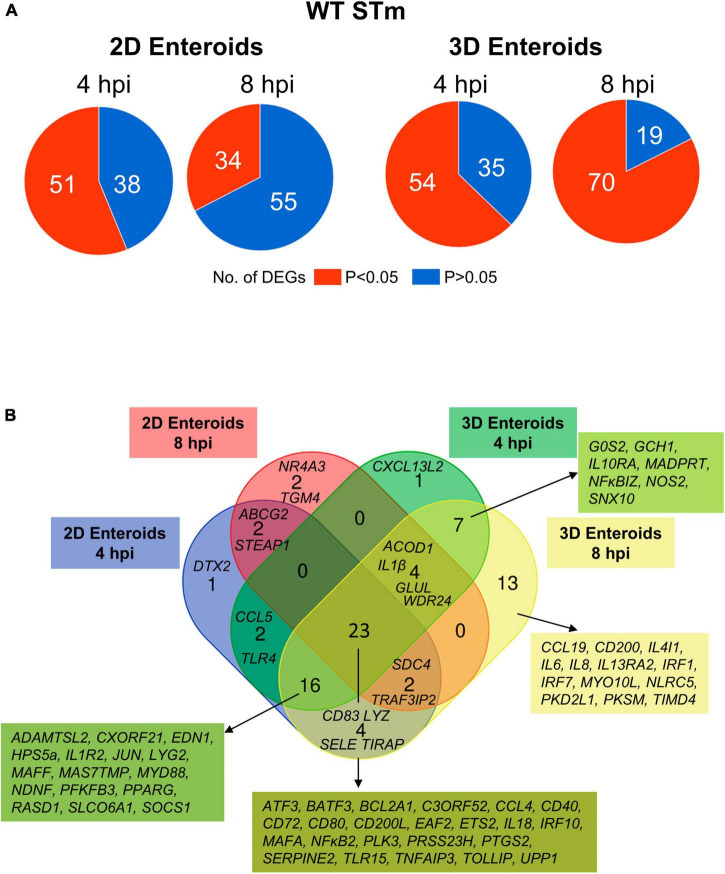
Innate immune response increase with time in WT STm infected 3D enteroids. **(A)** Pie charts indicating number of statistically significant, (*P* < 0.05) and non-significant (*P* > 0.05) DEGs in WT STm infected 2D enteroids (*n* = 3) and 3D enteroids (*n* = 5) at 4 hpi and 8 hpi compared to their time-matched, respective controls. DEGs with a significant difference (*P* < 0.05) and a fold change of ≥ 1.5 were identified. **(B)** Venn diagram showing common and unique DEGs across each enteroid model and timepoint.

Next, the statistically significant DEGs (fold change ≥ 1.5, *P* < 0.05 compared to their respective time-matched controls) was analyzed in 2D and 3D enteroids infected with WT STm at 4 and 8 hpi. STm infected 2D enteroids differentially expressed 51 genes at 4 hpi, decreasing to 34 genes by 8 hpi (Figure 7A). In contrast, the number of DEGs in WT STm infected 3D enteroids was 54 at 4 hpi rising to 70 at 8 hpi (Figure 7A). There were 23 DEGs in common between 2D and 3D enteroids at both time-points (Figure 7B). This core set of genes are involved in the regulation of immune responses, *ATF3*, *BATF3*, *ETS2*, *IRF10*, *PTGS2, MAFA, NF*κ*B2, TNFAIP3*, effector functions, *CCL4*, *CD200L*, *CD40*, *CD72*, *CD80*, *IL18*, and TLR signaling, *EAF2, TLR15, TOLLIP.* The second largest set of common genes (16 DEGs) was shared between 2D enteroids at 4 hpi and 3D enteroids at 4 and 8 hpi. These genes are involved in regulating immune responses (*JUN, MAFF, PPARG, RASD1, SOCS1)*, TLR/IL1β signaling *(MyD88, IL1R2)*, and effector functions (*LYG2*, *TLR4)* while *TLR4* and the chemokine, *CCL5*, were differentially expressed at 4 hpi in both models.

The number of DEGs that were uniquely expressed in one or the other model differed substantially, with the 2D enteroids expressing 5 unique genes; specifically, those encoding a regulator of Notch signaling (*DTX2)*, a membrane transporter (*ABCG2*), a metalloreductase (*STEAP1*), a nuclear receptor (*NR4A3*), and transglutaminase (*TGM4*). In contrast, the 3D enteroids uniquely expressed 21 genes upon STm infection with a majority associated with immune cell functions. The seven genes shared between 4 and 8 hpi are involved in regulating inflammation (*IL10RA, IL13RA2, NF*κ*BIZ*), bactericidal activity (*GCH1, NOS2*), membrane trafficking in endosomes (*SNX10*), and apoptosis (*GOS2*). By 8 hpi, genes involved in the regulation of interferon (IFN) and IFN-inducible genes (*IRF1*, *IRF7*, *NLRC5*) and macrophage and DC activities (*CCL19*, *CD200*, *IL4IL*, *TIMD4*) were found to be differentially expressed.

**FIGURE 8:**
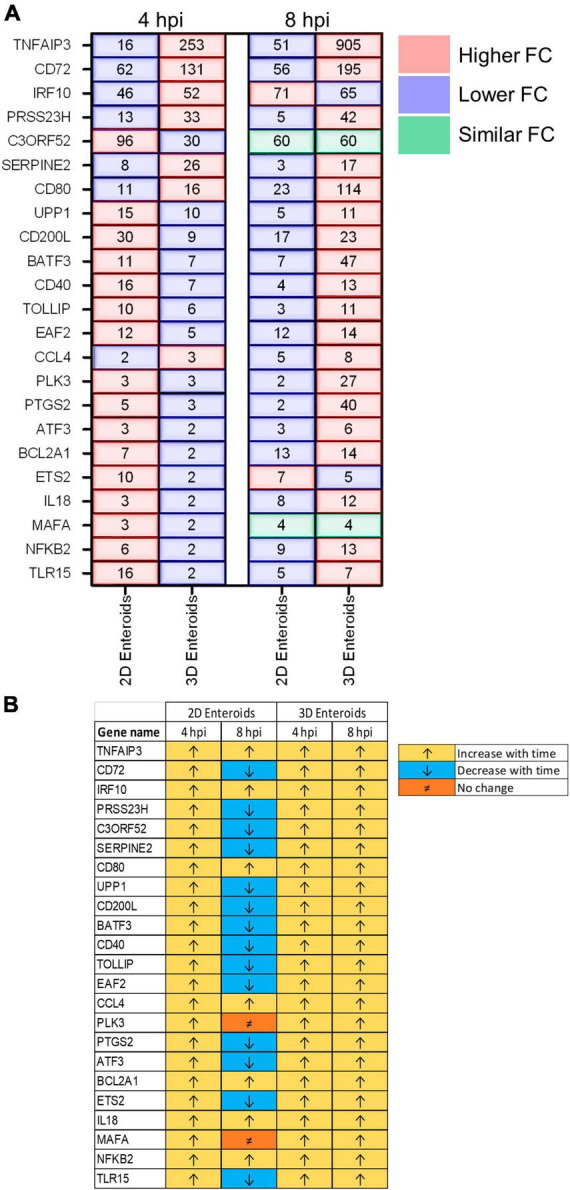
Lamina propria cells temporally govern the response to WT STm infection. **(A)** A comparison of the fold change levels of the common DEGs between the enteroid models demonstrates increased expression levels in 2D enteroids compared to 3D enteroids at 4 hpi. At 8 hpi, the expression levels of the common DEG are higher in 3D enteroids compared to 2D enteroids. **(B)** The expression levels of a majority of the common DEGs decrease with time in WT STm infected 2D enteroids and increase with time in 3D enteroids. Fold change values represent the mean of 3 (2D) or 5 (3D) independent experiments, relative to their time-matched respective controls.

The temporal changes in the fold change levels of the 23 common DEGs demonstrates that the vast majority were expressed at higher levels in the 2D enteroids compared to the 3D enteroids at 4 hpi (Figure 8A and Supplementary Material 2). In contrast, at 8 hpi the majority of the common genes had higher fold change levels in the 3D enteroids compared to 2D enteroids. The magnitude of the fold change for DEGs in 2D enteroids did not exceed 96 (*C3ORF52*), whereas in 3D enteroids three common DEGs, possibly involved in inhibiting inflammatory responses, were expressed at a fold change ranging from 114 to 905 (*CD72*, *CD80, TNFAIP3*). In addition, the magnitude of the fold change of all 23 common genes increased with time in 3D enteroids whereas 13 of the common genes decrease with time in 2D enteroids (Figure 8B). Overall, the data suggests that 2D and 3D enteroids have differing temporal responses to STm infection.

### 3.5. Chicken enteroids mount an innate immune response to non-invasive *Salmonella*

**FIGURE 9:**
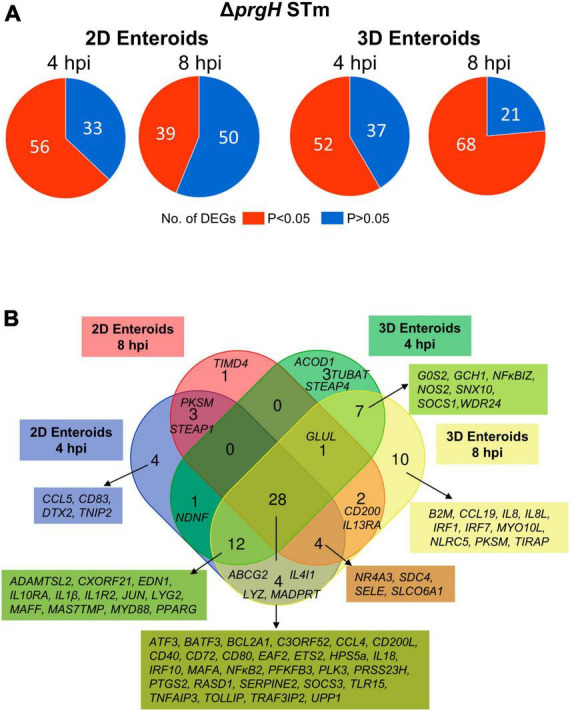
Chicken enteroids mount an innate immune response to non-invasive STm. **(A)** Pie charts indicating the number of statistically significant (*P* < 0.05) and non-significant (*P* > 0.05) DEGs in STm infected 2D enteroids (*n* = *3*) and 3D enteroids (*n* = *5*) at 4 hpi and 8 hpi, compared to their time-matched, respective controls. DEGs with a significant difference (*P* < 0.05) and a fold change of ≥ 1.5 were identified. **(B)** Venn diagram showing common and unique DEGs across each enteroid model and timepoint.

To characterize the effects of a non-invasive STm strain on innate immune responses, 2D and 3D enteroids were infected with the isogenic Δ*prgH* mutant of STm strain 4/74. At 4 and 8 hpi, 56 and 39 genes were differentially expressed in 2D enteroids at *P-*values < 0.05, fold change of ≥ 1.5, respectively (Figure 9A). In 3D enteroids, infections with the Δ*prgH* STm strain differentially affected 52 genes at 4 hpi at fold change of > 2 and *P-*values < 0.05, increasing to 68 genes by 8 hpi (Figure 9A). Analysis of the DEGs across the models and time-points indicated a core set of 28 common genes (Figure 9B). Similar to WT STm infection, genes involved in the regulation of immune responses (*ATF3*, *BATF3*, *ETS2*, *IRF9*, *NF*κ*B2, MAFA, TNFAIP3*), effector functions, (*CCL4*, *CD200L*, *CD40*, *CD72*, *CD80*, *IL18)* and TLR signaling, (*EAF2, TLR15, TRAF3IP2, TOLLIP*) were upregulated after Δ*prgH* STm infection in both models (Supplementary Material 2). An additional four genes were upregulated in Δ*prgH* STm infection and were involved in the regulation of glycolysis (*PFKFB3*) and cytokine signaling (*SOCS3*), and intracellular signaling (*RASD1*, *TRAF3IP2*). When disentangling the innate responses between the models, 8 and 20 genes were differentially expressed only in 2D and 3D enteroids, respectively. Similar to the response to WT STm infection, Δ*prgH* STm infected 2D enteroids regulated *CCL5* and *DTX2* at 4 hpi and genes involved in the regulation of antigen presentation (*CD83*), and inhibition of NFκB activation (*NFAIP2*). At both time-points, a gene involved in cell-cell junctions (*STEAP1*) and a serine protease (*PRSS23*) were upregulated and by 8 hpi, only one DEG, *TIMD4*, encoding a phosphatidylserine receptor for apoptotic cells, was specifically regulated in 2D enteroids. The shared genes in 3D enteroids were involved in nitric oxide synthesis (*GCH1, NOS2*), negative regulation of inflammation (*NF*κ*BIZ*, *SOCS1*), the mTOR signaling pathway (*WDR24*), and membrane trafficking in endosomes (*SNX10*).

**FIGURE 10:**
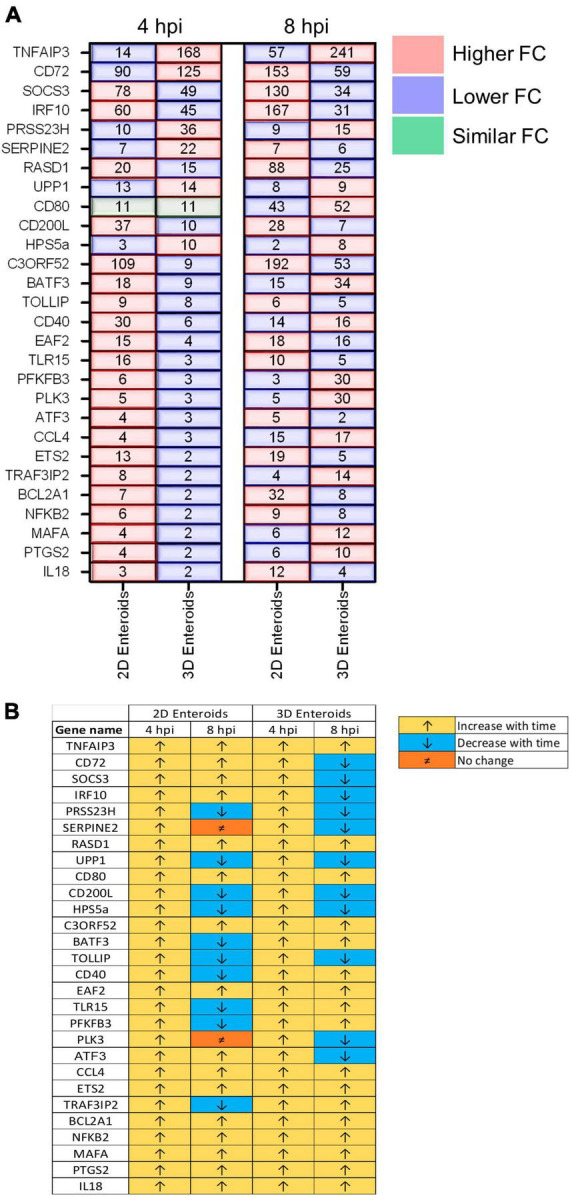
Δ*prgH* STm induces a rapid but brief innate immune response in chicken enteroids. **(A)** Fold change difference in the common DEGs in Δ*prgH* STm infected 2D and 3D enteroids at 4 hpi and 8 hpi. **(B)** The innate immune responses decrease with time in Δ*prgH* STm infected 2D and 3D enteroids. Fold change values represent the mean of 3 (2D) or 5 (3D) independent experiments, relative to their time-matched respective controls.

Fold change comparisons of the common genes demonstrates the expression levels for most genes were higher in 2D enteroids compared to 3D enteroids at 4 hpi (Figure 10A). However, by 8 hpi the expression levels for 15 out 28 common genes were higher in 2D enteroids compared to 3D enteroids. In contrast to infection with WT STm, when a differential response was detected between 2D and 3D enteroids over time, treatment with the invasive deficient Δ*prgH* STm strain resulted in the downregulation of almost half of the common DEGs in both 2D and 3D enteroids (Figure 10B). The data indicates that bacterial invasion is not required for 2D and 3D enteroids to mount an early response to STm, but invasion is required in 3D enteroids to increase the responses over-time.

### 3.6. Transcriptional regulation of innate immune genes remain unaffected by bacterial invasion

**FIGURE 11:**
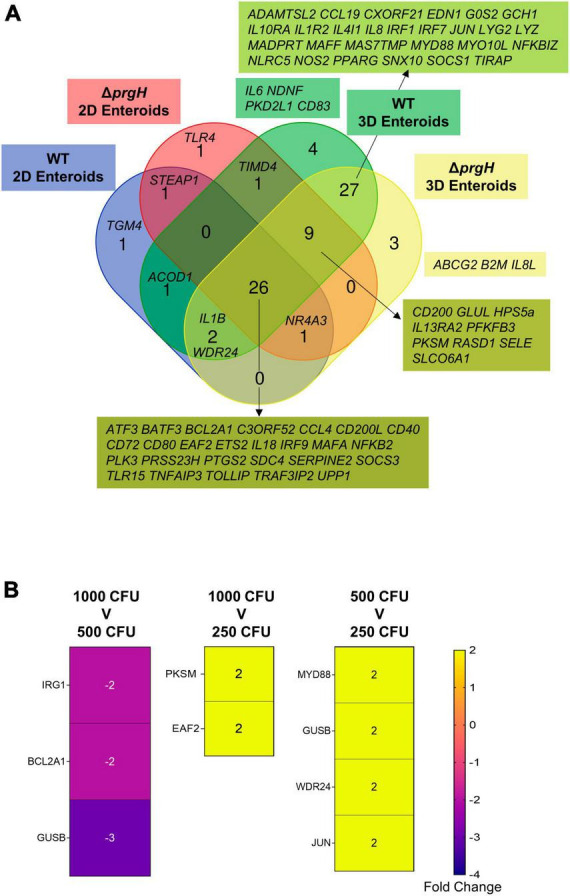
Transcriptional regulation of innate immune responses does not require bacterial invasion in chicken enteroids. **(A)** Venn diagram showing the DEGs that are altered by WT or Δ*prgH* STm infected 2D and 3D enteroids at 4 hpi and 8 hpi. **(B)** Heat maps of the fold change difference in the DEGs (Fold change ≥ 1.5, *P* < 0.05) between 1,000, 500, and 250 CFU of WT STm infected 3D enteroids demonstrates that the transcriptional regulation of innate immune genes is not significantly affected by bacterial burden. Fold change values represent the mean of 3 independent experiments.

When comparing the innate immune responses between 2D and 3D enteroids, a majority of the DEGs were common to both models when infected with the invasive WT or non-invasive Δ*prgH* STm. We next analyzed the contribution of the bacterial load to innate responses by comparing the DEGs (fold change of ≥ 1.5 and *P* < 0.05) in WT and Δ*prgH* STm infected enteroids at the late stage of infection, 8 hpi, when we observed numerous cells infected with WT STm in both models. Irrespective of STm strain, 26 DEGs were common across each model demonstrating the transcriptional regulation of these genes is independent of bacterial invasion (Figure 11). Few DEGs were uniquely upregulated by either WT or Δ*prgH* STm, independent of the enteroid model. WT and Δ*prgH* STm infected 2D enteroids shared 1 common gene (*STEAP1*) while each strain regulated 1 specific gene each, *TGM4* and *TLR4*. WT and Δ*prgH* STm infection of 3D enteroids shared 27 common genes while WT STm infection regulated 4 genes, (*CD83, IL6, NDNF, PKD2L1*) and Δ*prgH* STm regulated 3 genes (*ABCG2, B2M, IL8L*). STRING analysis reveals that the common DEGs at 8 hpi are linked to KEGG pathways involved in the intestinal immune network for IgA production and C-type lectin receptors signaling pathways (Supplementary Figure 3). To examine the contributions of lamina propria cells to signaling pathways, STRING analysis was carried out using the common DEGs across each model along with the common genes shared between WT and Δ*prgH* STm infected 3D enteroids at 8 hpi (Supplementary Figure 3). Key KEGG pathways such as Toll-like and NOD-like receptor signaling pathways, Cytosolic DNA-sensing pathway and Cytokine-cytokine receptor interaction were regulated in 3D enteroids.

To determine the effects of bacterial burden on the innate immune responses of lamina propria cells, we infected 3D enteroids with 1,000, 500 and 250 CFU of WT STm for 3 h and analyzed the mRNA expression levels using Fluidigm Biomark high-throughput qPCR. Few genes were significantly differentially regulated, with a fold change of ≥ 1.5 and *P-*values < 0.05, between the different doses of WT STm (Figure 11B). Moreover, the fold change of these DEGs was low. This demonstrates that the burden of bacteria does not significantly alter the transcriptional regulation of innate immune genes in chicken 3D enteroids and further supports the role lamina propria cells have on the regulation of responses to *Salmonella*.

## 4. Discussion

Currently, intestinal enteroids represent the gold standard *in vitro* models for studying host-pathogen interactions. In contrast to mammalian enteroids, the inner core of chicken 3D enteroids is solid and contains all cells that are present in the lamina propria because the isolated intact villi become enclosed to develop into 3D structures. In contrast, our 2D model contains all subsets of epithelial cells, few IELs and a layer of mesenchymal cells representing the lamina basalis. Chicken 2D and 3D enteroids offer valuable model systems in which to disentangle the interplay between epithelial/mesenchymal cells (2D) and epithelial/mesenchymal/lamina propria cells (3D) to reveal the cellular interplay that highly affects the overall response to danger signals including STm.

Previously, we demonstrated that WT STm can adhere and enter 3D enteroids in a manner associated with actin remodeling (Nash et al., 2021). In the current study, we used the same invasive WT STm and an isogenic invasion deficient Δ*prgH* mutant strain and demonstrated that the latter was adherent and immunogenic but did not invade chicken 2D and 3D enteroids. We observed that WT STm remodeled F-actin and disrupted tight junctions based on altered localization of ZO-1, whereas these phenotypes were not seen with the Δ*prgH* STm strain or after treatment with *Salmonella* LPS. ZO-1 associates with tight junction proteins, claudins and occludins, required to maintain cellular polarity and the paracellular barrier that allows selective passage of certain components (Odenwald et al., 2017). By altering ZO-1 expression, *Salmonella* increases the permeability of the epithelial layer which in turn enables invasive and otherwise non-invasive bacteria to enter the host tissue (Boyle et al., 2006). This process is known to be dependent on Type 3 secretion system-1 (T3SS-1) and a number of the effector proteins it injects, including SopB, SopE, SopE2, and SipA (Boyle et al., 2006). As the Δ*prgH* mutation abolishes the function of T3SS-1, the absence of disruption of ZO-1 localization upon infection with this strain is consistent with expectations. Much of the research on this topic has relied on mammalian cell lines and models and further work is required to understand the permeability properties of tight junctions in chickens and consequences of disruption of these by STm. Overall, our data are consistent with the known role of T3SS-1 and its effector proteins in *Salmonella* interactions with mammalian intestinal epithelial cells *in vitro* and *in vivo* (Jepson et al., 2000; Boyle et al., 2006; Lhocine et al., 2015).

The chicken 2D and 3D enteroids represent promising and versatile models to study host-pathogen interactions. In this study, we examine the response of 2D and 3D enteroids to LPS and STm infection using Fluidigm qPCR array to measure the mRNA expression levels of 89 innate immune related genes. These genes were selected based on their shared expression after infection of chickens or immune cells with *Salmonella*, *Campylobacter* and *Eimeria* or constituents thereof (Borowska et al., 2019). In contrast to 3D enteroids, 2D enteroids express more genes post-LPS treatment. Comparisons between the models found that after STm infection, a set of common genes was expressed in a temporal fashion in each model. For instance, in WT STm infected enteroids, the expression levels of 60% of the common genes increased with time in 3D enteroids but these genes decreased in 2D enteroids. In Δ*prgH* infected enteroids, 42% of the common genes decreased with time in both models. As the microbiota produce copious amounts of LPS, and to prevent unwanted stimulation and maintain homeostasis, TLR4 expression is restricted to the basal region of mammalian epithelial cells (Price et al., 2018). In this study, we cultured 2D enteroids on plastic plates and in contrast to 2D enteroids grown on Matrigel coated transwell inserts, epithelial barrier integrity could not be measured (Orr et al., 2021). The 2D conformation may not be completely tight and some leakage of LPS to the basolateral sides of the cells may have occurred. Furthermore, the chicken 3D enteroid resembles the natural conformation of the epithelium and prevents access of LPS to the basolateral sides of the cells similar to the *in vivo* situation. This would make the TLRs inaccessible. Therefore, we would recommend to either grow the 2D enteroids on a transwell insert and measure epithelial barrier integrity prior to LPS exposure or use the 3D conformation. Although in the current study, intracellular bacterial replication was not quantified, we demonstrated that varying the size of the bacterial inoculum induced no significant changes in gene expression levels and therefore the differences in temporal gene expression may be related to the presence of lamina propria cells in 3D enteroids and the structural differences between the models. Future studies involving use of fluorescence dilution may allow replication of *Salmonella* to be measured at the single cell level, including to understand the fate of STm in different types of infected cells. Overall, the differences in temporal gene expression may be related to the presence of lamina propria cells in 3D enteroids and the structural differences between the models.

Interestingly, the invasion deficient Δ*prgH* STm strain was found in close contact with the apical surface of epithelial cells in both models and induced an early immune response at a magnitude similar to WT STm treatment. The close physical association is likely mediated by bacterial adhesins such as fimbriae, flagella and outer membrane proteins (Stones and Krachler, 2016). In addition, day-old chicks are highly susceptible to *Salmonella* infection, usually leading to death (Withanage et al., 2005). In this study, we utilized the small intestines from ED18 embryos, therefore taken together both these attributes may be driving an immune response independent of bacterial invasion. Further research is necessary to examine the differences in the immune response between enteroids-derived from older chickens and how different STm virulence factors affect immune responses.

*In vitro* and *in vivo* chicken studies have illuminated how the immune system in tissues and cells respond to *Salmonella.* We also found similarities between our models and these studies. In 3D enteroids, the phagocyte chemoattractant genes, *IL8* (*CXCLi1*) and *IL8L* (*CXCLi2*) and transcription factors, *IRF1* and *IRF7*, were induced by STm infection (Poh et al., 2008; Setta et al., 2012). *IRF1* and *IRF7* are upregulated in chicken heterophils stimulated with *S*. Enteritidis leading to the induction of IFNs (Chiang et al., 2008; Kogut et al., 2012). *IL4l1*, a putative anti-inflammatory gene expressed by myeloid cells was upregulated in 3D enteroids (Marquet et al., 2010). IL4IL was the most inducible gene found in chicken cecum post-*S*. Enteritidis infection (Elsheimer-Matulova et al., 2020). Genes upregulated in 2D and/or 3D enteroids, such as *IL6*, *IL1*β, *IL10RA*, *IL18*, *MyD88*, were found to be upregulated in the spleen and caecum of STm infected chickens (Dar et al., 2019, 2022; Ahmad et al., 2023). Genes such as *RASD1, SOSC3*, *TOLLIP*, that are involved in the negative regulation of downstream TLR4 signaling, NFκB activation and cytokine activities, were upregulated across all models. In contrast both *TOLLIP* and *RASD1* were found to be downregulated in *S*. Enteritidis challenged chickens (Tsai et al., 2010; Sun et al., 2019). This discrepancy can be caused by the model or by the strain of *Salmonella* used. DEGs in both 2D and 3D enteroids are linked to pathways associated with Toll-like receptor signaling, intestinal immune network IgA production, Cytosolic DNA sensing, and Cytokine-receptor interaction. These pathways are similarly regulated in chicken intestines after STm infection (Wang et al., 2019; Khan and Chousalkar, 2020; Dar et al., 2022). Overall, most key features of the immune response against STm *in vivo* could be replicated in 2D and 3D enteroids. Future single-cell RNA sequencing studies will help to fully resolve the contributions and cross-talk between the different epithelial, mesenchymal and immune cells to STm in 2D and 3D enteroids.

Nitric oxide (NO) production is a vital host-defense mechanism against microbial pathogens in mononuclear phagocytes. *NOS2*, was downregulated in 3D enteroids irrespective of the STm strain. *S*. Enteritidis suppresses *NOS2* expression in HD11 cells and experimentally infected chickens (He et al., 2012, 2018; Cazals et al., 2022). In a recent immunometabolic kinome peptide array analysis, *S*. Enteritidis but not *S*. Heidelberg or *S*. Senftenberg infected HD11 cells inhibited NO production and reduced glycolysis which together indicate macrophage polarization from pro-inflammatory, M1, to anti-inflammatory, M2, state (He et al., 2023). Our data indicates that chicken 3D enteroids provide a suitable *ex vivo* intestinal model to examine the relationship between macrophage polarization and immuno-metabolism during infection with different *Salmonella* strains.

## 5. Conclusion

In conclusion, the chicken 2D and 3D enteroids allowed for the first time a description of the distinct innate immune responses exhibited by epithelial cells and lamina propria cells. The enteroid models replicated several observations demonstrated after *in vivo* infection of chickens with *Salmonella*, including the alteration of tight junctions and the induction of inflammatory responses. The chicken enteroid models offers many advantages over other models to reduce animal use in the study of host-pathogen interactions.

## Data availability statement

The original contributions presented in this study are included in the article/Supplementary material, further inquiries can be directed to the corresponding authors.

## Ethics statement

The animal study was approved by The Roslin Institute’s Animal Welfare and Ethical Review Board. The study was conducted in accordance with the local legislation and institutional requirements.

## Author contributions

KS, TN, and LV conceptualized the study. LV secured funding to undertake the work and supervised the project. KS, TN, SS, DB, and JM performed the experiments and analyzed the data with support of PV, MS, and LV. KS wrote the manuscript supported by SS, PV, MS, and LV. All authors read and approved the final manuscript.
