# Supplementary material for: Disentangling the innate immune responses of intestinal epithelial cells and lamina propria cells to Salmonella Typhimurium infection in chickens
Source: Front Microbiol. 2023 Oct 3;14:1258796. doi: 10.3389/fmicb.2023.1258796 (PMC10579587; doi:10.3389/fmicb.2023.1258796)
Supplement: Supplementary file 3 [file Presentation_1.PPTX]

## Slide 1
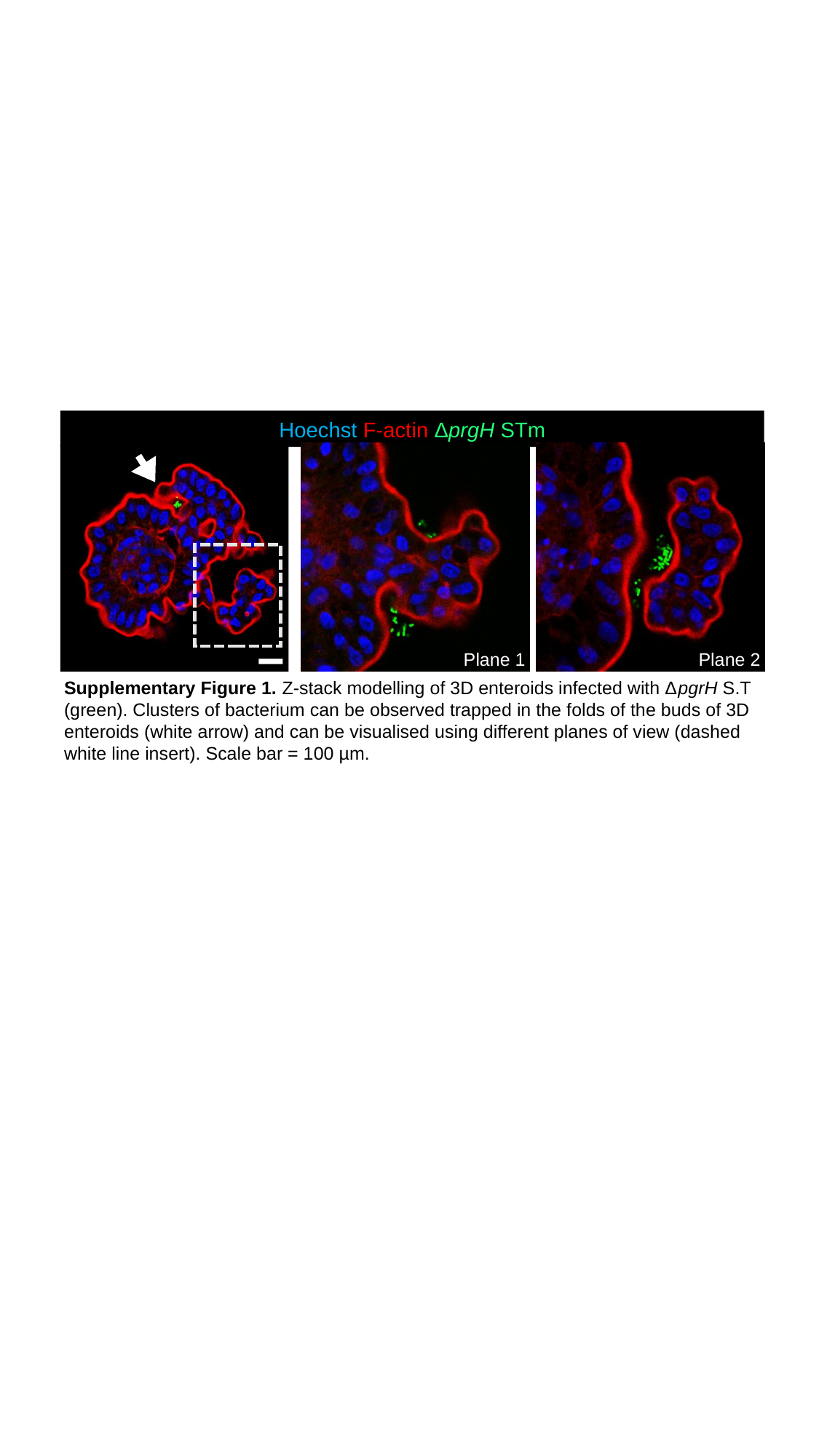

Hoechst F-actin ΔprgH STm
Plane 1
Plane 2
Supplementary Figure 1. Z-stack modelling of 3D enteroids infected with ΔpgrH S.T (green). Clusters of bacterium can be observed trapped in the folds of the buds of 3D enteroids (white arrow) and can be visualised using different planes of view (dashed white line insert). Scale bar = 100 µm.
