# Supplementary material for: Disentangling the innate immune responses of intestinal epithelial cells and lamina propria cells to Salmonella Typhimurium infection in chickens
Source: Front Microbiol. 2023 Oct 3;14:1258796. doi: 10.3389/fmicb.2023.1258796 (PMC10579587; doi:10.3389/fmicb.2023.1258796)
Supplement: Supplementary file 4 [file Presentation_2.PPTX]

## Slide 1
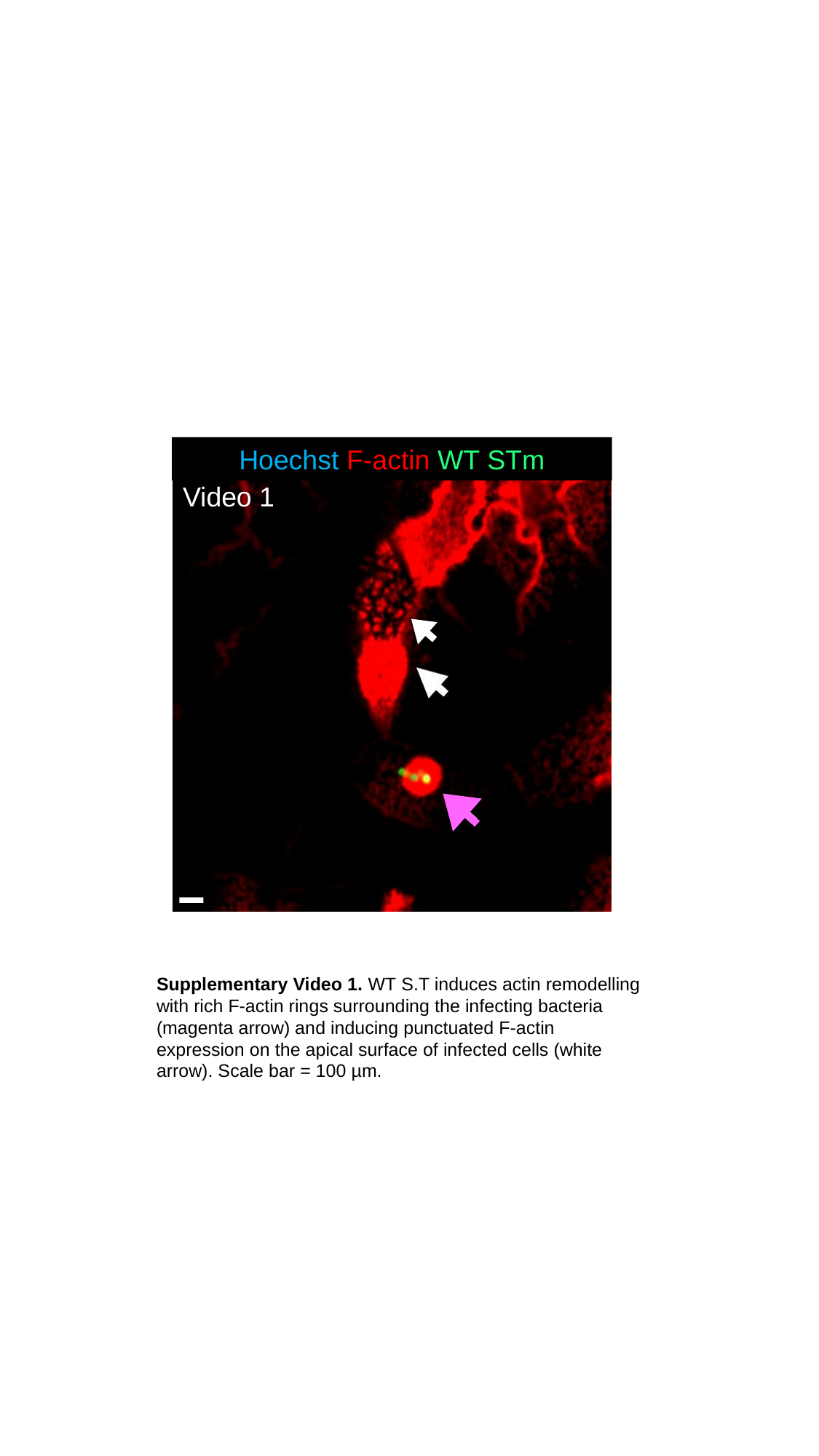

Hoechst F-actin WT STm
Video 1
Video
Supplementary Video 1. WT S.T induces actin remodelling with rich F-actin rings surrounding the infecting bacteria (magenta arrow) and inducing punctuated F-actin expression on the apical surface of infected cells (white arrow). Scale bar = 100 µm.
