# Supplementary material for: Disentangling the innate immune responses of intestinal epithelial cells and lamina propria cells to Salmonella Typhimurium infection in chickens
Source: Front Microbiol. 2023 Oct 3;14:1258796. doi: 10.3389/fmicb.2023.1258796 (PMC10579587; doi:10.3389/fmicb.2023.1258796)
Supplement: Supplementary file 5 [file Presentation_3.PPTX]

## Slide 1
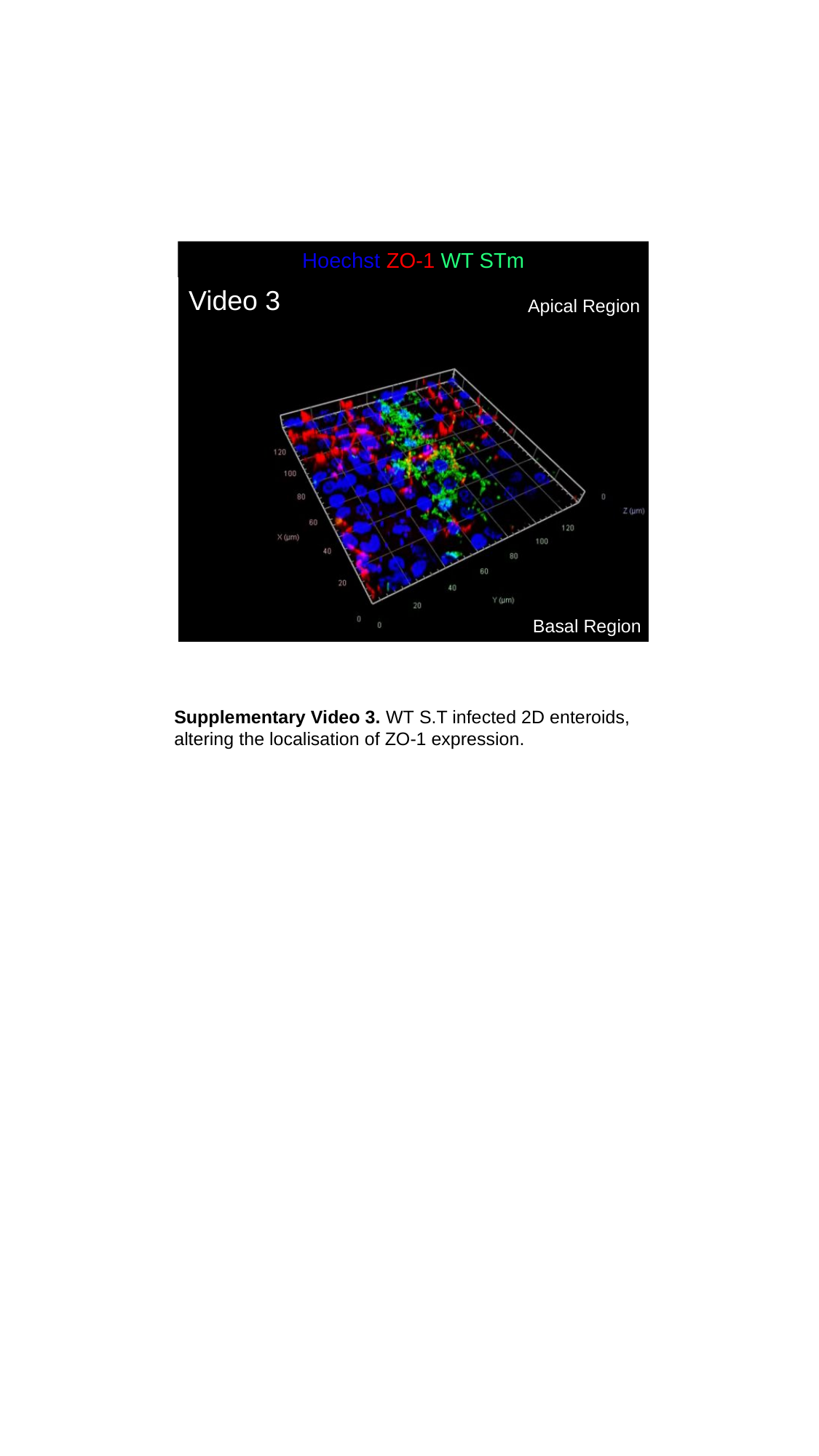

Hoechst ZO-1 WT STm
Video 3
Apical Region
Basal Region
Supplementary Video 3. WT S.T infected 2D enteroids, altering the localisation of ZO-1 expression.
