# Supplementary material for: Disentangling the innate immune responses of intestinal epithelial cells and lamina propria cells to Salmonella Typhimurium infection in chickens
Source: Front Microbiol. 2023 Oct 3;14:1258796. doi: 10.3389/fmicb.2023.1258796 (PMC10579587; doi:10.3389/fmicb.2023.1258796)
Supplement: Supplementary file 6 [file Presentation_4.PPTX]

## Slide 1
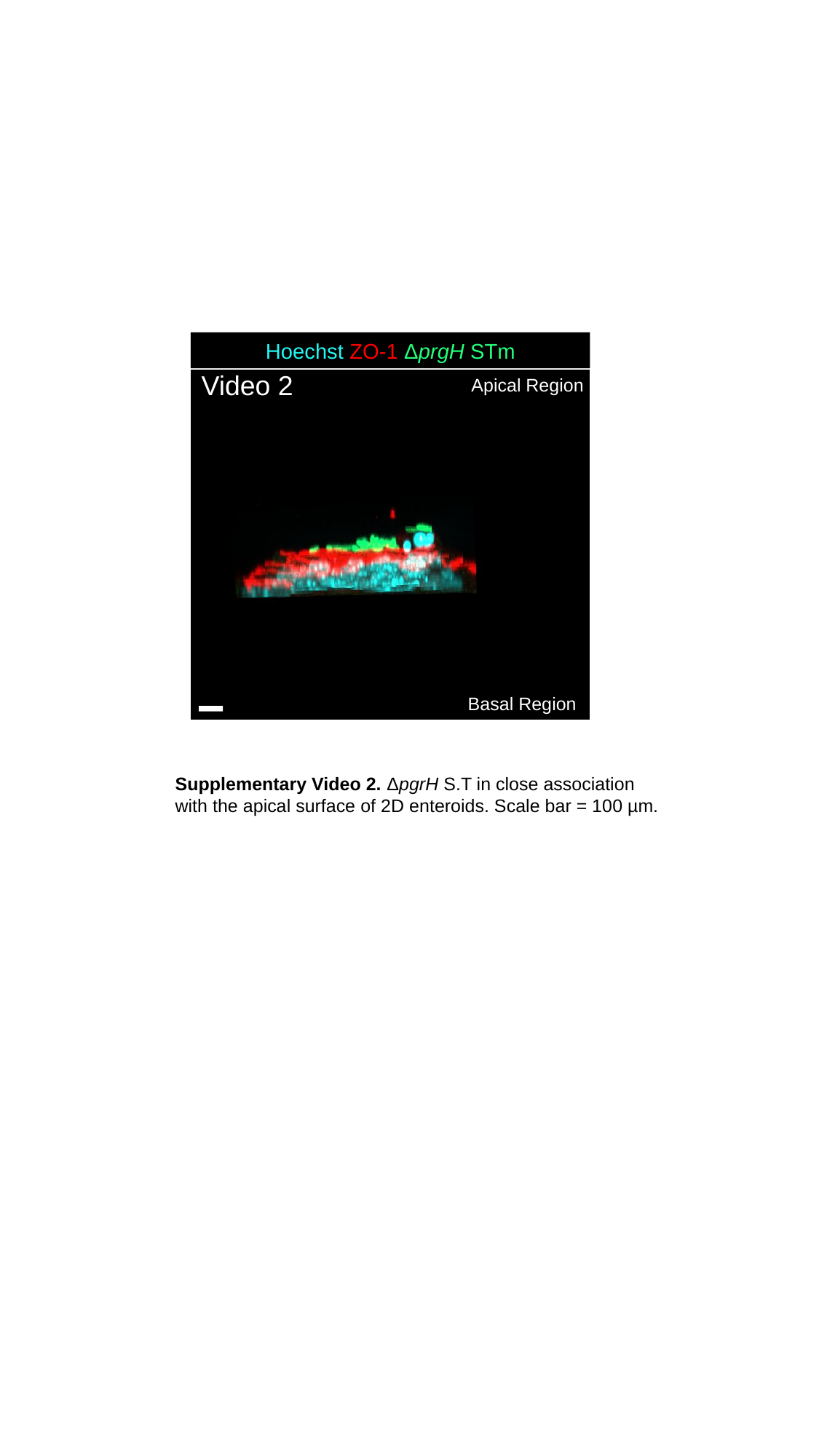

Hoechst ZO-1 ΔprgH STm
Video 2
Apical Region
Basal Region
Supplementary Video 2. ΔpgrH S.T in close association with the apical surface of 2D enteroids. Scale bar = 100 µm.
Apical surface of 3D enteroid
