# Supplementary material for: Disentangling the innate immune responses of intestinal epithelial cells and lamina propria cells to Salmonella Typhimurium infection in chickens
Source: Front Microbiol. 2023 Oct 3;14:1258796. doi: 10.3389/fmicb.2023.1258796 (PMC10579587; doi:10.3389/fmicb.2023.1258796)
Supplement: Supplementary file 7 [file Presentation_5.PPTX]

## Slide 1
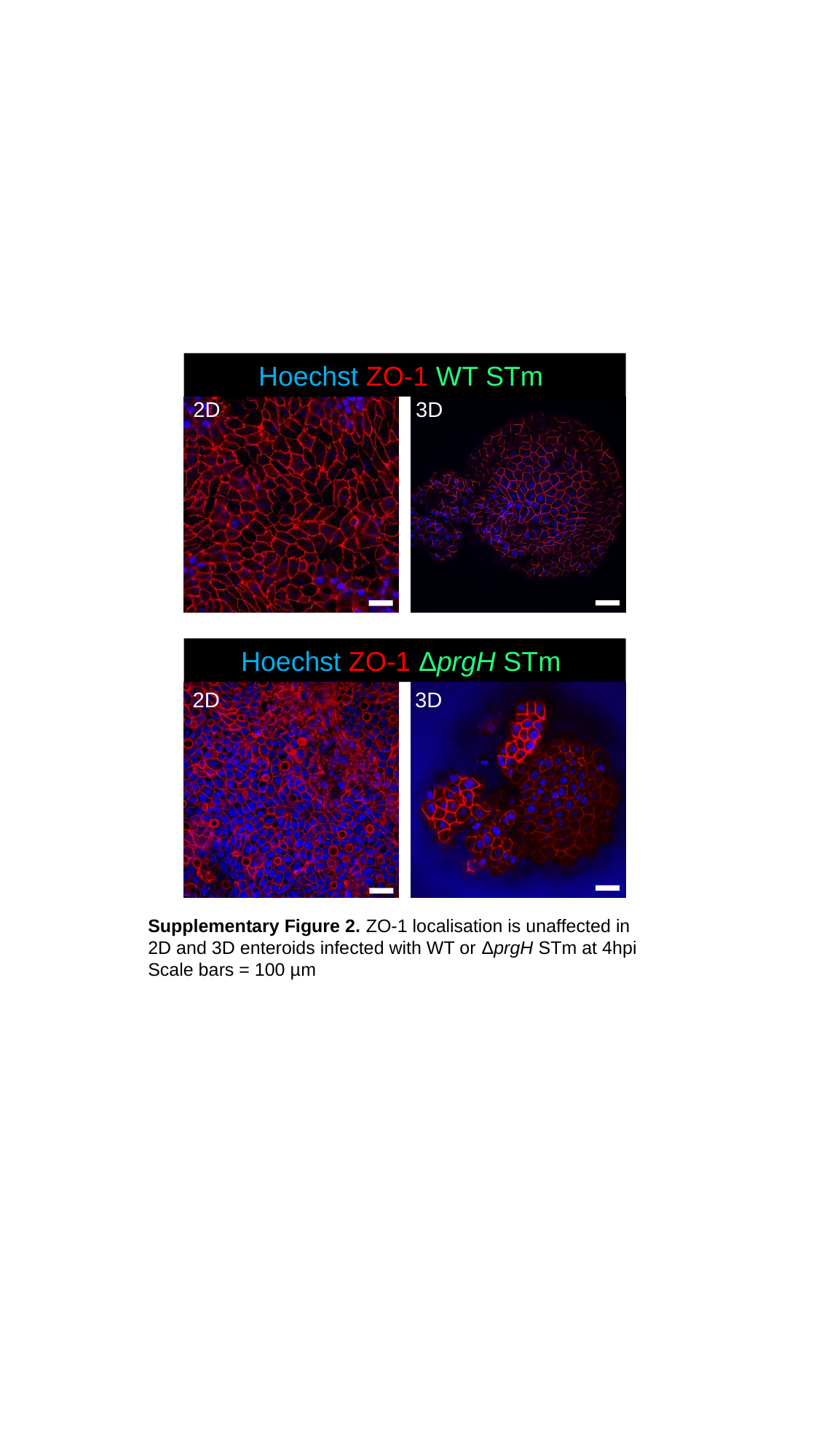

Hoechst ZO-1 WT STm
3D
2D
Hoechst ZO-1 ΔprgH STm
3D
2D
Supplementary Figure 2. ZO-1 localisation is unaffected in 2D and 3D enteroids infected with WT or ΔprgH STm at 4hpi Scale bars = 100 µm
