# Supplementary material for: Disentangling the innate immune responses of intestinal epithelial cells and lamina propria cells to Salmonella Typhimurium infection in chickens
Source: Front Microbiol. 2023 Oct 3;14:1258796. doi: 10.3389/fmicb.2023.1258796 (PMC10579587; doi:10.3389/fmicb.2023.1258796)
Supplement: Supplementary file 8 [file Presentation_6.PPTX]

## Slide 1
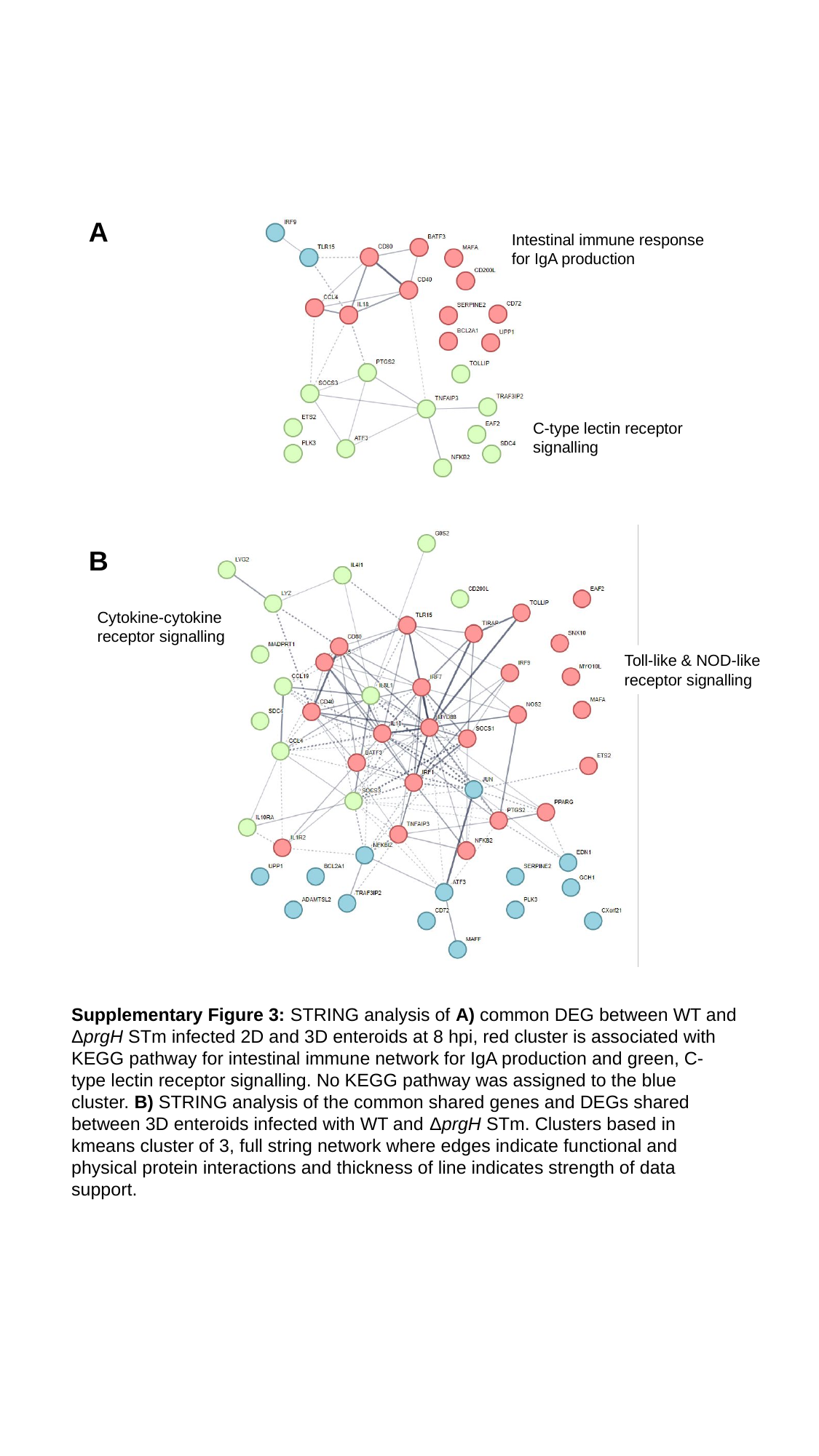

A
Intestinal immune response for IgA production
C-type lectin receptor signalling
B
Cytokine-cytokine receptor signalling
Toll-like & NOD-like receptor signalling
Supplementary Figure 3: STRING analysis of A) common DEG between WT and ΔprgH STm infected 2D and 3D enteroids at 8 hpi, red cluster is associated with KEGG pathway for intestinal immune network for IgA production and green, C-type lectin receptor signalling. No KEGG pathway was assigned to the blue cluster. B) STRING analysis of the common shared genes and DEGs shared between 3D enteroids infected with WT and ΔprgH STm. Clusters based in kmeans cluster of 3, full string network where edges indicate functional and physical protein interactions and thickness of line indicates strength of data support.
